# Crystal structure of (*S*)-5-chloro-*N*-({2-oxo-3-[4-(3-oxomorpholin-4-yl)phen­yl]oxazolidin-5-yl}meth­yl)thio­phene-2-carboxamide

**DOI:** 10.1107/S2056989017017819

**Published:** 2018-01-01

**Authors:** Jie Shen, Gu-Ping Tang, Xiu-Rong Hu

**Affiliations:** a Chemistry Department, Zhejiang University, Hangzhou, Zhejiang 310028, People’s Republic of China; b Institute of Chemical Biology and Pharmaceutical Chemistry, Zhejiang University, Hangzhou, Zhejiang 310028, People’s Republic of China

**Keywords:** crystal structure, rivaroxaban, Hirshfeld surface, hydrogen bonds

## Abstract

The asymmetric unit of the crystal of the title compound (common name rivaroxaban), C_19_H_18_ClN_3_O_5_, contains two rivaroxaban mol­ecules with different conformations; the C—C—N—C torsion angles between the oxazolidine and thio­phene rings are −171.1 (7) and −106.8 (9)° in the two independent mol­ecules. In the crystal, classical N—H⋯O hydrogen bonds and weak C—H⋯O hydrogen bonds link the mol­ecules into a three-dimensional supra­molecular architecture.

## Abstract 2

The asymmetric unit of the crystal of the title compound contains two rivaroxaban mol­ecules with different conformations.

## Chemical context   

At present, the incidence of thromboic disease is extremely high; this is mainly caused by vascular endothelial injury, increased blood coagulation, increased platelet number and decreased anti­coagulant activity (Lassila, 2012 ▸). In anti­coagulants, warfarin and heparin have dominated the market, but they have some defects such as making making patients bleed easily and be prone to thrombocytopenia and osteoporosis (Mega & Carreras, 2012 ▸). In recent years, factor Xa inhibitors, the new type of anti­coagulant drugs, have received more and more attention, and rivaroxaban is a representative drug of factor Xa inhibitors (Goel & Srivathsan, 2012 ▸).
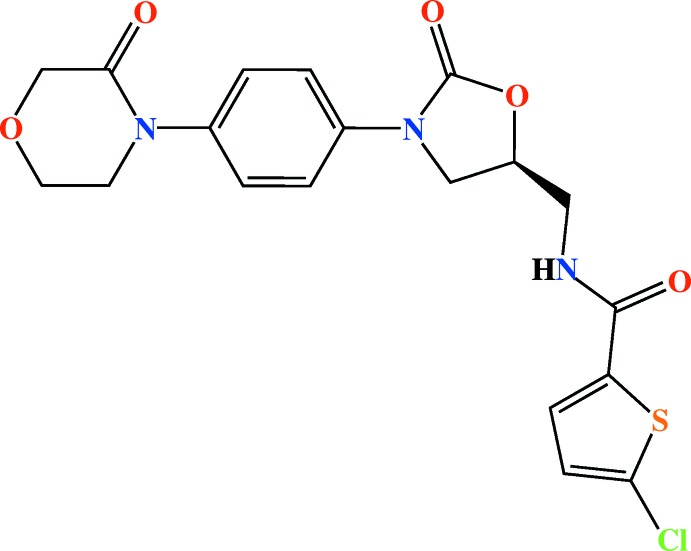


Rivaroxaban is a novel oral direct factor Xa inhibitor that inhibits factor Xa selectively, thereby prolongs prothrombin time and reduces thrombin generation (Ansell, 2007 ▸). It does not have a direct effect on thrombin but it inhibits the formation of thrombin by inhibiting factor Xa activity, which impedes the formation of fibrin in turn and ultimately inhibits thrombus formation and enlargement (Perzborn *et al.*, 2005 ▸). In 2011, rivaroxaban was approved by the US Food and Drug Administration (FDA) for the prevention of stroke or systemic embolism in patients with non-valvular atrial fibrillation. The patent WO2007039132 (Ludescher *et al.*, 2012 ▸) concerned crystalline form I, form II, form III, the amorphous form, the hydrate, the NMP solvate and the THF clathrate of rivaroxaban. However, there are few reports on the single-crystal structure of rivaroxaban. As part of our ongoing structural studies of pharmaceutical compounds, the crystal structure of rivaroxaban is presented here.

## Structural commentary   

**Figure 1:**
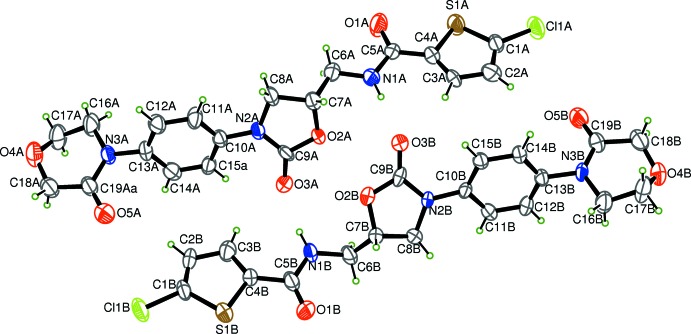
The mol­ecular structure of the title compound,showing the atom-labelling scheme and displacement ellipsoids at the 50% probability level. H atoms are shown as small circles of arbitrary radii.

The mol­ecular structure of the title compound is shown in Fig. 1 ▸. The asymmetric unit contains two mol­ecules with different conformations. In the *N*-methyl­formamide moieties of mol­ecules *A* and *B*, the C7—C6—N1—C5 torsion angles are −171.1 (7) and −106.8 (9)°, respectively (Table 1 ▸). The oxazolidine ring of mol­ecule *A* is almost planar [the maximum deviation is 0.048 (6) Å for the O2*A* atom], whereas the oxazolidine ring of mol­ecule *B* displays an envelope conformation with atom C8*B* as the flap. The morpholine rings of the two mol­ecules display similar twisted boat conformations. Atoms O4 and C17 deviate from the C16/N3/C19/C18 mean plane by 0.230 (2) and 0.517 (2) Å, respectively, in mol­ecule *A* and by 0.290 (2) and 0.489 (2) Å in mol­ecule *B*.

## Supra­molecular features   

**Figure 2:**
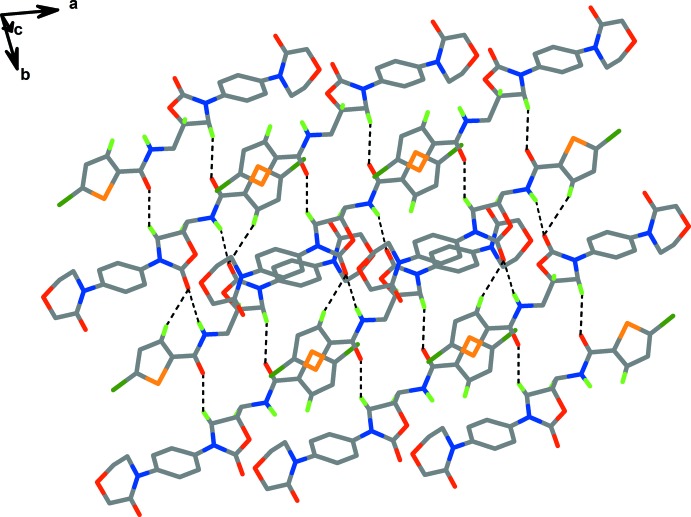
The supermolecular structure showing the inter­molecular inter­actions (Table 2 ▸) as dashed lines.

In the crystal, N—H⋯O hydrogen bonds (Table 2 ▸, Fig. 2 ▸) link the independent mol­ecules *A* and *B* into dimers, and weak C—H⋯O hydrogen bonds link the dimers to form a three-dimensional supra­molecular architecture (Table 2 ▸).

## Hirshfeld surface analysis   

**Figure 3:**
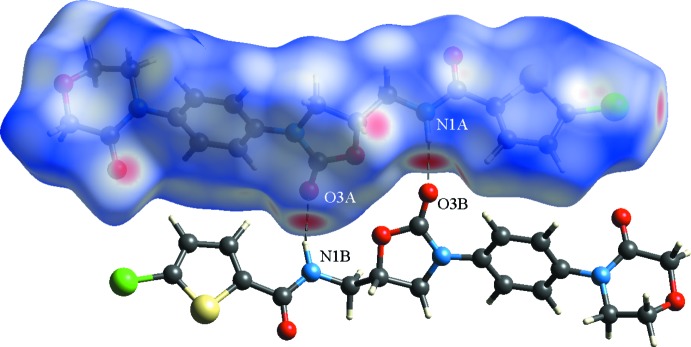
Plots of *d*_norm_ mapped on the Hirshfeld surfaces of the title compound showing the N—H⋯O hydrogen bonds.

The Hirshfeld surface of a mol­ecule in a crystal is constructed by calculating the spherical atom electron densities. On the *d*_norm_ surface, when inter­molecular contacts are shorter than the sum of van der Waals radii, they are highlighted in red, longer contacts in blue and contacts around the sum of van der Waals radii in white. The Hirshfeld surface analyses and two-dimensional fingerprint plots for the title compound were generated by *CrystalExplorer* (Wolff *et al.*, 2013 ▸), and are illustrated in Figs. 3 ▸ and 4 ▸, respectively.

The light-red spots on the Hirshfeld surface are the results of N—H⋯O, C—H⋯O and C—Cl⋯O inter­actions (Fig. 3 ▸). The H⋯H contacts, which comprise 27% of the total Hirshfeld surface area, appear in the central region of the fingerprint plot (Fig. 3 ▸ *b*). The O⋯H/H⋯O inter­actions (22.4%), which are the most significant inter­molecular inter­actions and link the mol­ecular dimers into infinite chains along the *b* axis, appear as two obvious spikes (Fig. 3 ▸ *c*). At the top left (*d*_i_ < *d*_e_) and bottom right (*d*_i_ > *d*_e_) of the fingerprint plot, there are characteristic ‘wings’ that are identified resulting from the C⋯H/H⋯C inter­actions (18.7%) shown in Fig. 3 ▸ *d*.

## Synthesis and crystallization   

The crude product was supplied by the Zhejiang Huadong Pharmaceutical Co., Ltd. It was recrystallized from methanol solution, giving colourless crystals suitable for X-ray diffraction.

## Refinement   

Crystal data, data collection and structure refinement details are summarized in Table 3 ▸. N-bound atoms H1*A* and H1*B* were found in difference-Fourier maps, but placed in calculated positions with N—H = 0.86 Å and refined as riding with *U*_iso_(H) = 1.2*U*_eq_(N). All other H atoms were placed in calculated positions with C—H = 0.93–0.98 Å and included in the refinement in a riding model, with *U*_iso_(H) = 1.2 or 1.5*U*_eq_(carrier atom).

## Supplementary Material

Crystal structure: contains datablock(s) global, I. DOI: 10.1107/S2056989017017819/xu5913sup1.cif

Structure factors: contains datablock(s) I. DOI: 10.1107/S2056989017017819/xu5913Isup2.hkl

Supporting information file. DOI: 10.1107/S2056989017017819/xu5913Isup3.cml

CCDC reference: 1810879

Additional supporting information:  crystallographic information; 3D view; checkCIF report

## Figures and Tables

**Figure 4:**
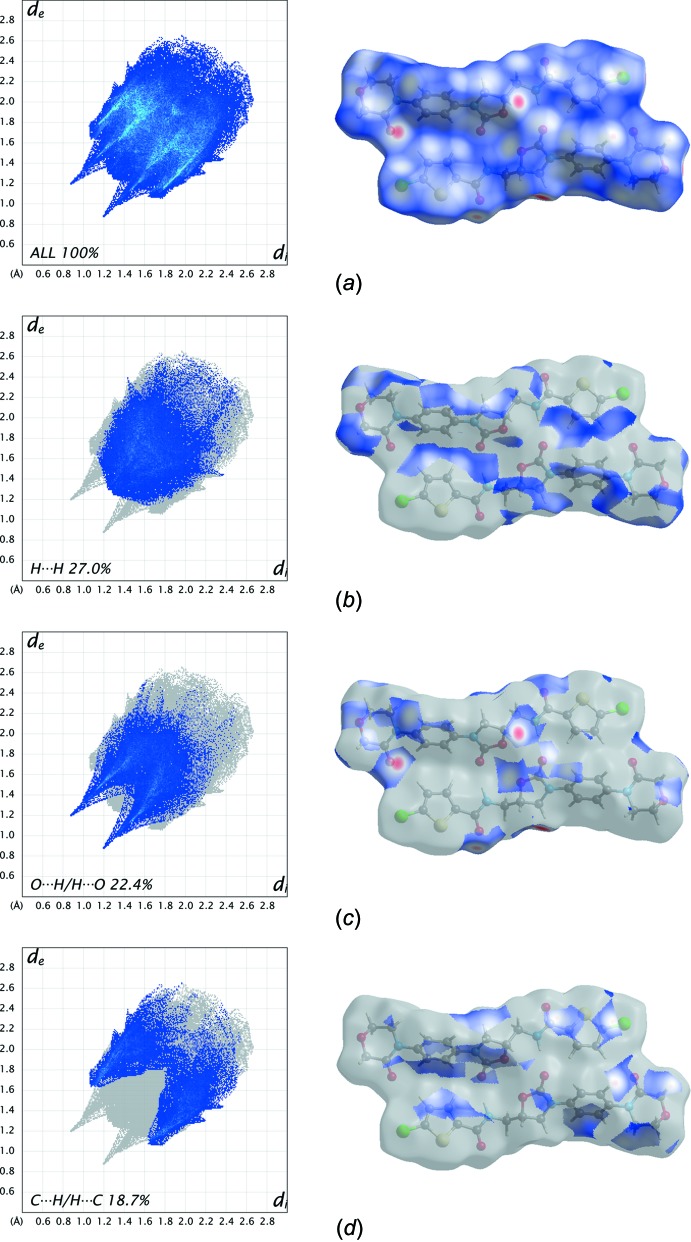
The two-dimensional fingerprint of title compound showing contributions from different contacts.

**Table 1:** Selected torsion angles (°)

C3*A*—C4*A*—C5*A*—O1*A*	172.0 (10)	C7*A*—C6*A*—N1*A*—C5*A*	−171.1 (7)
C3*B*—C4*B*—C5*B*—O1*B*	−169.2 (9)	O1*B*—C5*B*—N1*B*—C6*B*	10.8 (13)
O1*A*—C5*A*—N1*A*—C6*A*	−4.0 (13)	C4*B*—C5*B*—N1*B*—C6*B*	−166.0 (7)
C4*A*—C5*A*—N1*A*—C6*A*	176.0 (6)	C7*B*—C6*B*—N1*B*—C5*B*	−106.8 (9)

**Table 2:** Hydrogen-bond geometry (Å, °)

*D*—H⋯*A*	*D*—H	H⋯*A*	*D*⋯*A*	*D*—H⋯*A*
N1*A*—H1*A*⋯O3*B*	0.86	2.16	3.008 (11)	169
N1*B*—H1*B*⋯O3*A*	0.86	2.22	3.016 (11)	153
C3*A*—H3*A*⋯O3*B*	0.93	2.48	3.357 (11)	157
C6*B*—H6*B*1⋯O5*A* ^i^	0.97	2.41	3.227 (10)	141
C7*A*—H7*A*⋯O5*B* ^ii^	0.98	2.41	3.288 (8)	149
C8*A*—H8*A*2⋯O1*B* ^iii^	0.97	2.52	3.459 (11)	163
C8*B*—H8*B*1⋯O1*A* ^iv^	0.97	2.15	2.961 (12)	140

Symmetry codes: (i) 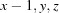; (ii) 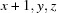; (iii) 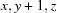; (iv) 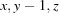.

**Table 3:** Experimental details

Crystal data	
Chemical formula	C_19_H_18_ClN_3_O_5_S
*M*_r_	435.87
Crystal system, space group	Triclinic, *P*1
Temperature (K)	296
*a*, *b*, *c* (Å)	9.0184 (6), 10.9980 (8), 11.2386 (8)
α, β, γ (°)	63.426 (2), 74.414 (3), 78.144 (2)
*V* (Å^3^)	955.56 (12)
*Z*	2
Radiation type	Mo *K*α
μ (mm^−1^)	0.35
Crystal size (mm)	0.39 × 0.27 × 0.06

Data collection	
Diffractometer	Rigaku R-AXIS RAPID
Absorption correction	Multi-scan (*ABSCOR*; Higashi, 1995 ▸)
*T*_min_, *T*_max_	0.868, 0.979
No. of measured, independent and observed [*I* > 2σ(*I*)] reflections	8375, 6267, 3981
*R*_int_	0.043
(sin θ/λ)_max_ (Å^−1^)	0.617

Refinement	
*R*[*F* ^2^ > 2σ(*F* ^2^)], *wR*(*F* ^2^), *S*	0.045, 0.149, 1.00
No. of reflections	6267
No. of parameters	524
No. of restraints	3
H-atom treatment	H-atom parameters constrained
Δρ_max_, Δρ_min_ (e Å^−3^)	0.33, −0.48
Absolute structure	Flack (1983 ▸), 2519 Friedel pairs
Absolute structure parameter	−0.07 (13)

Computer programs: *PROCESS-AUTO* (Rigaku, 2006 ▸), *CrystalStructure* (Rigaku, 2007 ▸), *SHELXS97* and *SHELXL97* (Sheldrick, 2008 ▸), *ORTEP-3 for Windows* (Farrugia, 2012 ▸) and *WinGX* and *DIAMOND* (Brandenburg & Putz, 2006 ▸).

## supplementary crystallographic information

### Crystal data

**Table:**

C_19_H_18_ClN_3_O_5_S	*Z* = 2
*M**_r_* = 435.87	*F*(000) = 452
Triclinic, *P*1	*D*_x_ = 1.515 Mg m^−^^3^
Hall symbol: P 1	Mo *K*α radiation, λ = 0.71073 Å
*a* = 9.0184 (6) Å	Cell parameters from 6182 reflections
*b* = 10.9980 (8) Å	θ = 3.3–27.5°
*c* = 11.2386 (8) Å	µ = 0.35 mm^−^^1^
α = 63.426 (2)°	*T* = 296 K
β = 74.414 (3)°	Platelet, colorless
γ = 78.144 (2)°	0.39 × 0.27 × 0.06 mm
*V* = 955.56 (12) Å^3^	

### Data collection

**Table:**

Rigaku R-AXIS RAPID diffractometer	6267 independent reflections
Radiation source: rotating anode	3981 reflections with *I* > 2σ(*I*)
Graphite monochromator	*R*_int_ = 0.043
Detector resolution: 10.00 pixels mm^-1^	θ_max_ = 26.0°, θ_min_ = 3.3°
ω scans	*h* = −11→10
Absorption correction: multi-scan (ABSCOR; Higashi, 1995)	*k* = −13→13
*T*_min_ = 0.868, *T*_max_ = 0.979	*l* = −13→12
8375 measured reflections	

### Refinement

**Table:**

Refinement on *F*^2^	Hydrogen site location: inferred from neighbouring sites
Least-squares matrix: full	H-atom parameters constrained
*R*[*F*^2^ > 2σ(*F*^2^)] = 0.045	*w* = 1/[σ^2^(*F*_o_^2^) + (0.0576*P*)^2^ + 0.9688*P*] where *P* = (*F*_o_^2^ + 2*F*_c_^2^)/3
*wR*(*F*^2^) = 0.149	(Δ/σ)_max_ < 0.001
*S* = 1.00	Δρ_max_ = 0.33 e Å^−^^3^
6267 reflections	Δρ_min_ = −0.47 e Å^−^^3^
524 parameters	Extinction correction: SHELXL97 (Sheldrick, 2008), Fc^*^=kFc[1+0.001xFc^2^λ^3^/sin(2θ)]^-1/4^
3 restraints	Extinction coefficient: 0.0153 (18)
Primary atom site location: structure-invariant direct methods	Absolute structure: Flack (1983), 2519 Friedel pairs
Secondary atom site location: difference Fourier map	Absolute structure parameter: −0.07 (13)

### Special details

**Table:**

Geometry. All esds (except the esd in the dihedral angle between two l.s. planes) are estimated using the full covariance matrix. The cell esds are taken into account individually in the estimation of esds in distances, angles and torsion angles; correlations between esds in cell parameters are only used when they are defined by crystal symmetry. An approximate (isotropic) treatment of cell esds is used for estimating esds involving l.s. planes.
Refinement. Refinement of F^2^ against ALL reflections. The weighted R-factor wR and goodness of fit S are based on F^2^, conventional R-factors R are based on F, with F set to zero for negative F^2^. The threshold expression of F^2^ > 2sigma(F^2^) is used only for calculating R-factors(gt) etc. and is not relevant to the choice of reflections for refinement. R-factors based on F^2^ are statistically about twice as large as those based on F, and R- factors based on ALL data will be even larger.

### Fractional atomic coordinates and isotropic or equivalent isotropic displacement parameters (Å^2^)

**Table:**

	*x*	*y*	*z*	*U*_iso_*/*U*_eq_	
C1A	−0.1583 (9)	1.0666 (8)	0.2283 (8)	0.0439 (19)	
C2A	−0.0913 (9)	0.9526 (9)	0.2067 (9)	0.055 (2)	
H2A	−0.1318	0.9137	0.1654	0.066*	
C3A	0.0484 (9)	0.9019 (8)	0.2560 (8)	0.049 (2)	
H3A	0.1097	0.8251	0.2496	0.059*	
C4A	0.0845 (9)	0.9721 (8)	0.3113 (8)	0.044 (2)	
C5A	0.2153 (9)	0.9561 (8)	0.3737 (8)	0.046 (2)	
C6A	0.4500 (8)	0.8320 (8)	0.4481 (8)	0.0536 (19)	
H6A1	0.4173	0.8227	0.5410	0.064*	
H6A2	0.5024	0.9143	0.3946	0.064*	
C7A	0.5590 (6)	0.7108 (6)	0.4437 (6)	0.0432 (14)	
H7A	0.5844	0.7152	0.3515	0.052*	
C8A	0.7087 (8)	0.6965 (8)	0.4921 (7)	0.0468 (18)	
H8A1	0.7986	0.7055	0.4193	0.056*	
H8A2	0.7049	0.7645	0.5259	0.056*	
C9A	0.5857 (9)	0.4996 (8)	0.6187 (8)	0.0426 (18)	
C10A	0.8327 (8)	0.5027 (8)	0.6745 (7)	0.0371 (17)	
C11A	0.9704 (8)	0.5597 (9)	0.6184 (8)	0.051 (2)	
H11A	0.9842	0.6319	0.5327	0.061*	
C12A	1.0901 (9)	0.5086 (9)	0.6909 (8)	0.052 (2)	
H12A	1.1832	0.5479	0.6538	0.063*	
C13A	1.0706 (9)	0.4001 (8)	0.8169 (7)	0.0405 (19)	
C14A	0.9315 (9)	0.3423 (9)	0.8685 (8)	0.048 (2)	
H14A	0.9183	0.2673	0.9521	0.058*	
C15A	0.8124 (9)	0.3937 (8)	0.7984 (7)	0.0425 (18)	
H15A	0.7192	0.3546	0.8350	0.051*	
C16A	1.2171 (11)	0.4524 (11)	0.9410 (11)	0.067 (3)	
H16A	1.1206	0.5014	0.9664	0.080*	
H16B	1.2822	0.5185	0.8665	0.080*	
C17A	1.2969 (10)	0.3788 (11)	1.0610 (9)	0.065 (3)	
H17A	1.3259	0.4449	1.0837	0.078*	
H17B	1.2270	0.3213	1.1393	0.078*	
C18A	1.3869 (11)	0.1916 (9)	1.0115 (10)	0.059 (2)	
H18A	1.3534	0.1208	1.1008	0.071*	
H18B	1.4791	0.1531	0.9671	0.071*	
C19A	1.2629 (9)	0.2279 (9)	0.9317 (8)	0.048 (2)	
C1B	1.0264 (9)	−0.0628 (8)	0.7509 (8)	0.0427 (19)	
C2B	0.9694 (9)	0.0466 (9)	0.7726 (9)	0.052 (2)	
H2B	1.0163	0.0829	0.8121	0.062*	
C3B	0.8282 (10)	0.1024 (10)	0.7290 (9)	0.054 (2)	
H3B	0.7724	0.1812	0.7352	0.065*	
C4B	0.7817 (9)	0.0297 (8)	0.6767 (8)	0.0427 (19)	
C5B	0.6395 (9)	0.0457 (9)	0.6254 (8)	0.047 (2)	
C6B	0.3859 (8)	0.1766 (7)	0.5957 (8)	0.056 (2)	
H6B1	0.3174	0.2091	0.6606	0.068*	
H6B2	0.3573	0.0878	0.6168	0.068*	
C7B	0.3595 (7)	0.2715 (6)	0.4581 (6)	0.0417 (13)	
H7B	0.4197	0.2367	0.3911	0.050*	
C8B	0.1891 (8)	0.2963 (7)	0.4524 (9)	0.052 (2)	
H8B1	0.1633	0.2406	0.4154	0.062*	
H8B2	0.1238	0.2786	0.5414	0.062*	
C9B	0.2926 (8)	0.5022 (9)	0.3553 (7)	0.0397 (18)	
C10B	0.0478 (8)	0.5025 (8)	0.2993 (7)	0.0347 (16)	
C11B	−0.0894 (9)	0.4413 (8)	0.3481 (8)	0.0444 (19)	
H11B	−0.1005	0.3637	0.4297	0.053*	
C12B	−0.2088 (9)	0.4918 (9)	0.2796 (8)	0.049 (2)	
H12B	−0.2986	0.4478	0.3144	0.059*	
C13B	−0.1964 (9)	0.6065 (8)	0.1604 (8)	0.0391 (18)	
C14B	−0.0649 (8)	0.6727 (8)	0.1093 (7)	0.0392 (17)	
H14B	−0.0578	0.7520	0.0292	0.047*	
C15B	0.0591 (8)	0.6212 (8)	0.1778 (7)	0.0367 (17)	
H15B	0.1485	0.6658	0.1425	0.044*	
C16B	−0.3420 (11)	0.5655 (11)	0.0280 (11)	0.069 (3)	
H16C	−0.3988	0.4912	0.0998	0.083*	
H16D	−0.2424	0.5266	−0.0061	0.083*	
C17B	−0.4295 (10)	0.6406 (12)	−0.0841 (10)	0.065 (3)	
H17C	−0.3674	0.7069	−0.1616	0.078*	
H17D	−0.4543	0.5772	−0.1126	0.078*	
C18B	−0.5312 (10)	0.8161 (9)	−0.0125 (9)	0.056 (2)	
H18C	−0.6237	0.8480	0.0378	0.067*	
H18D	−0.5027	0.8917	−0.0996	0.067*	
C19B	−0.4012 (9)	0.7757 (9)	0.0650 (8)	0.0437 (19)	
N1A	0.3152 (7)	0.8431 (7)	0.3946 (7)	0.0498 (17)	
H1A	0.2999	0.7788	0.3769	0.060*	
N2A	0.7116 (7)	0.5601 (7)	0.5999 (6)	0.0372 (14)	
N3A	1.1862 (8)	0.3562 (7)	0.8972 (7)	0.0432 (15)	
N1B	0.5436 (7)	0.1595 (7)	0.6146 (7)	0.0532 (17)	
H1B	0.5767	0.2250	0.6192	0.064*	
N2B	0.1756 (6)	0.4401 (6)	0.3618 (6)	0.0368 (14)	
N3B	−0.3186 (7)	0.6567 (7)	0.0831 (6)	0.0409 (15)	
O1A	0.2326 (8)	1.0454 (7)	0.4062 (8)	0.084 (2)	
O2A	0.4876 (6)	0.5867 (5)	0.5382 (6)	0.0555 (15)	
O3A	0.5553 (7)	0.3836 (6)	0.6960 (6)	0.0604 (16)	
O4A	1.4274 (7)	0.2998 (7)	1.0281 (7)	0.0691 (19)	
O5A	1.2297 (8)	0.1426 (7)	0.9017 (6)	0.0673 (17)	
O1B	0.6129 (7)	−0.0440 (6)	0.6016 (7)	0.0673 (17)	
O2B	0.3984 (6)	0.4081 (6)	0.4212 (6)	0.0482 (13)	
O3B	0.3058 (7)	0.6245 (6)	0.3060 (6)	0.0541 (14)	
O4B	−0.5669 (7)	0.7076 (7)	−0.0354 (6)	0.0594 (16)	
O5B	−0.3806 (7)	0.8525 (6)	0.1100 (6)	0.0622 (16)	
S1A	−0.0530 (2)	1.1087 (2)	0.3076 (2)	0.0611 (6)	
S1B	0.9128 (2)	−0.1087 (2)	0.6818 (2)	0.0563 (6)	
Cl1A	−0.3274 (2)	1.1591 (2)	0.1863 (2)	0.0640 (6)	
Cl1B	1.1962 (2)	−0.1602 (2)	0.7917 (2)	0.0675 (7)	

### Atomic displacement parameters (Å^2^)

**Table:**

	*U*^11^	*U*^22^	*U*^33^	*U*^12^	*U*^13^	*U*^23^
C1A	0.037 (4)	0.045 (5)	0.042 (4)	−0.002 (4)	−0.016 (3)	−0.008 (4)
C2A	0.054 (5)	0.043 (5)	0.064 (5)	0.001 (4)	−0.017 (4)	−0.019 (4)
C3A	0.040 (4)	0.034 (4)	0.072 (5)	0.015 (3)	−0.030 (4)	−0.018 (4)
C4A	0.041 (5)	0.038 (5)	0.054 (4)	0.005 (4)	−0.019 (4)	−0.016 (4)
C5A	0.040 (4)	0.036 (4)	0.067 (5)	0.000 (3)	−0.023 (4)	−0.021 (4)
C6A	0.044 (4)	0.049 (4)	0.081 (5)	0.002 (3)	−0.028 (4)	−0.031 (4)
C7A	0.041 (3)	0.043 (3)	0.043 (3)	−0.002 (2)	−0.015 (3)	−0.013 (3)
C8A	0.039 (4)	0.044 (4)	0.050 (4)	0.001 (3)	−0.021 (3)	−0.008 (3)
C9A	0.056 (5)	0.025 (4)	0.048 (4)	0.014 (3)	−0.030 (4)	−0.013 (3)
C10A	0.034 (4)	0.041 (4)	0.042 (4)	0.006 (3)	−0.014 (3)	−0.023 (4)
C11A	0.032 (4)	0.064 (6)	0.047 (4)	−0.013 (4)	−0.011 (3)	−0.009 (4)
C12A	0.033 (4)	0.050 (5)	0.058 (5)	−0.004 (3)	−0.008 (4)	−0.010 (4)
C13A	0.033 (4)	0.049 (5)	0.042 (4)	0.006 (3)	−0.017 (3)	−0.020 (4)
C14A	0.052 (5)	0.042 (4)	0.047 (4)	0.001 (4)	−0.015 (4)	−0.016 (4)
C15A	0.038 (4)	0.048 (5)	0.044 (4)	−0.009 (3)	−0.017 (3)	−0.015 (4)
C16A	0.067 (6)	0.073 (6)	0.092 (7)	0.013 (5)	−0.050 (5)	−0.051 (6)
C17A	0.057 (5)	0.091 (7)	0.066 (6)	0.004 (5)	−0.027 (5)	−0.046 (5)
C18A	0.061 (5)	0.053 (6)	0.071 (5)	0.014 (4)	−0.032 (4)	−0.031 (5)
C19A	0.045 (4)	0.046 (5)	0.052 (4)	0.008 (4)	−0.021 (4)	−0.019 (4)
C1B	0.032 (4)	0.046 (5)	0.041 (4)	0.001 (3)	−0.016 (3)	−0.008 (4)
C2B	0.039 (4)	0.062 (6)	0.067 (5)	−0.003 (4)	−0.028 (4)	−0.029 (5)
C3B	0.052 (5)	0.060 (6)	0.065 (5)	0.000 (4)	−0.027 (4)	−0.033 (5)
C4B	0.034 (4)	0.037 (5)	0.057 (5)	−0.001 (3)	−0.021 (4)	−0.015 (4)
C5B	0.038 (4)	0.045 (5)	0.053 (5)	0.006 (4)	−0.019 (3)	−0.014 (4)
C6B	0.035 (4)	0.042 (4)	0.080 (5)	0.004 (3)	−0.025 (4)	−0.011 (4)
C7B	0.041 (3)	0.036 (3)	0.055 (4)	0.001 (2)	−0.024 (3)	−0.020 (3)
C8B	0.047 (4)	0.031 (4)	0.080 (5)	0.002 (3)	−0.033 (4)	−0.017 (4)
C9B	0.024 (3)	0.055 (5)	0.048 (4)	−0.011 (3)	−0.010 (3)	−0.024 (4)
C10B	0.027 (3)	0.035 (4)	0.050 (4)	−0.001 (3)	−0.016 (3)	−0.021 (4)
C11B	0.046 (4)	0.035 (4)	0.046 (4)	0.000 (3)	−0.021 (3)	−0.006 (3)
C12B	0.034 (4)	0.054 (5)	0.065 (5)	−0.009 (3)	−0.023 (4)	−0.021 (4)
C13B	0.036 (4)	0.037 (4)	0.053 (4)	0.006 (3)	−0.022 (3)	−0.023 (4)
C14B	0.036 (4)	0.038 (4)	0.043 (4)	0.000 (3)	−0.018 (3)	−0.011 (3)
C15B	0.032 (4)	0.035 (4)	0.038 (4)	−0.004 (3)	−0.010 (3)	−0.009 (3)
C16B	0.064 (5)	0.066 (6)	0.111 (8)	0.023 (5)	−0.054 (6)	−0.058 (6)
C17B	0.051 (5)	0.095 (7)	0.076 (6)	0.000 (5)	−0.031 (5)	−0.053 (6)
C18B	0.047 (5)	0.053 (5)	0.056 (5)	0.002 (4)	−0.020 (4)	−0.011 (4)
C19B	0.034 (4)	0.049 (5)	0.047 (4)	0.005 (3)	−0.013 (3)	−0.019 (4)
N1A	0.044 (4)	0.037 (4)	0.076 (4)	0.008 (3)	−0.027 (3)	−0.027 (3)
N2A	0.028 (3)	0.037 (3)	0.041 (3)	0.000 (2)	−0.014 (2)	−0.010 (3)
N3A	0.041 (3)	0.045 (4)	0.053 (4)	0.010 (3)	−0.026 (3)	−0.025 (3)
N1B	0.039 (4)	0.042 (4)	0.071 (4)	−0.001 (3)	−0.032 (3)	−0.008 (3)
N2B	0.034 (3)	0.035 (3)	0.049 (3)	−0.002 (2)	−0.024 (3)	−0.016 (3)
N3B	0.040 (3)	0.046 (4)	0.050 (4)	0.003 (3)	−0.028 (3)	−0.024 (3)
O1A	0.074 (4)	0.074 (5)	0.153 (6)	0.024 (3)	−0.068 (4)	−0.076 (5)
O2A	0.053 (3)	0.031 (3)	0.079 (4)	−0.002 (2)	−0.045 (3)	−0.003 (3)
O3A	0.060 (3)	0.041 (4)	0.082 (4)	−0.011 (3)	−0.039 (3)	−0.011 (3)
O4A	0.049 (4)	0.084 (5)	0.081 (4)	0.005 (3)	−0.030 (3)	−0.035 (4)
O5A	0.070 (4)	0.055 (4)	0.088 (4)	0.006 (3)	−0.036 (3)	−0.034 (3)
O1B	0.069 (4)	0.054 (4)	0.107 (5)	0.006 (3)	−0.047 (3)	−0.046 (4)
O2B	0.041 (3)	0.045 (3)	0.066 (3)	−0.004 (2)	−0.027 (2)	−0.020 (3)
O3B	0.057 (3)	0.037 (3)	0.071 (3)	−0.004 (2)	−0.035 (3)	−0.013 (3)
O4B	0.046 (3)	0.066 (4)	0.073 (4)	0.004 (3)	−0.034 (3)	−0.027 (3)
O5B	0.068 (4)	0.051 (4)	0.078 (4)	0.014 (3)	−0.037 (3)	−0.032 (3)
S1A	0.0506 (12)	0.0551 (14)	0.0912 (17)	0.0171 (10)	−0.0335 (12)	−0.0409 (14)
S1B	0.0468 (12)	0.0469 (13)	0.0783 (15)	0.0098 (9)	−0.0291 (10)	−0.0255 (12)
Cl1A	0.0391 (11)	0.0656 (16)	0.0746 (14)	0.0071 (10)	−0.0234 (11)	−0.0167 (12)
Cl1B	0.0391 (11)	0.0587 (15)	0.0823 (15)	0.0071 (10)	−0.0275 (11)	−0.0062 (12)

### Geometric parameters (Å, º)

**Table:**

C1A—C2A	1.371 (11)	C1B—S1B	1.703 (9)
C1A—Cl1A	1.704 (8)	C1B—Cl1B	1.722 (8)
C1A—S1A	1.707 (9)	C2B—C3B	1.408 (12)
C2A—C3A	1.420 (12)	C2B—H2B	0.9300
C2A—H2A	0.9300	C3B—C4B	1.361 (12)
C3A—C4A	1.315 (12)	C3B—H3B	0.9300
C3A—H3A	0.9300	C4B—C5B	1.491 (12)
C4A—C5A	1.471 (12)	C4B—S1B	1.714 (8)
C4A—S1A	1.733 (8)	C5B—O1B	1.213 (10)
C5A—O1A	1.239 (10)	C5B—N1B	1.344 (10)
C5A—N1A	1.344 (10)	C6B—N1B	1.456 (10)
C6A—N1A	1.456 (10)	C6B—C7B	1.480 (9)
C6A—C7A	1.496 (8)	C6B—H6B1	0.9700
C6A—H6A1	0.9700	C6B—H6B2	0.9700
C6A—H6A2	0.9700	C7B—O2B	1.460 (8)
C7A—O2A	1.451 (7)	C7B—C8B	1.518 (9)
C7A—C8A	1.540 (9)	C7B—H7B	0.9800
C7A—H7A	0.9800	C8B—N2B	1.449 (9)
C8A—N2A	1.447 (9)	C8B—H8B1	0.9700
C8A—H8A1	0.9700	C8B—H8B2	0.9700
C8A—H8A2	0.9700	C9B—O3B	1.222 (9)
C9A—O3A	1.217 (9)	C9B—N2B	1.340 (9)
C9A—O2A	1.351 (9)	C9B—O2B	1.348 (9)
C9A—N2A	1.353 (10)	C10B—C11B	1.390 (11)
C10A—C15A	1.367 (10)	C10B—C15B	1.402 (10)
C10A—C11A	1.370 (11)	C10B—N2B	1.404 (10)
C10A—N2A	1.425 (10)	C11B—C12B	1.370 (11)
C11A—C12A	1.399 (12)	C11B—H11B	0.9300
C11A—H11A	0.9300	C12B—C13B	1.365 (11)
C12A—C13A	1.379 (11)	C12B—H12B	0.9300
C12A—H12A	0.9300	C13B—C14B	1.371 (11)
C13A—C14A	1.386 (11)	C13B—N3B	1.453 (10)
C13A—N3A	1.429 (11)	C14B—C15B	1.405 (10)
C14A—C15A	1.379 (11)	C14B—H14B	0.9300
C14A—H14A	0.9300	C15B—H15B	0.9300
C15A—H15A	0.9300	C16B—N3B	1.465 (11)
C16A—N3A	1.448 (11)	C16B—C17B	1.492 (13)
C16A—C17A	1.516 (12)	C16B—H16C	0.9700
C16A—H16A	0.9700	C16B—H16D	0.9700
C16A—H16B	0.9700	C17B—O4B	1.413 (10)
C17A—O4A	1.382 (10)	C17B—H17C	0.9700
C17A—H17A	0.9700	C17B—H17D	0.9700
C17A—H17B	0.9700	C18B—O4B	1.439 (11)
C18A—O4A	1.413 (11)	C18B—C19B	1.523 (12)
C18A—C19A	1.500 (12)	C18B—H18C	0.9700
C18A—H18A	0.9700	C18B—H18D	0.9700
C18A—H18B	0.9700	C19B—O5B	1.225 (10)
C19A—O5A	1.240 (11)	C19B—N3B	1.323 (9)
C19A—N3A	1.369 (10)	N1A—H1A	0.8600
C1B—C2B	1.309 (11)	N1B—H1B	0.8600
			
C2A—C1A—Cl1A	126.4 (7)	O1B—C5B—N1B	123.9 (9)
C2A—C1A—S1A	112.0 (6)	O1B—C5B—C4B	119.9 (8)
Cl1A—C1A—S1A	121.6 (5)	N1B—C5B—C4B	116.1 (8)
C1A—C2A—C3A	110.7 (8)	N1B—C6B—C7B	115.4 (6)
C1A—C2A—H2A	124.6	N1B—C6B—H6B1	108.4
C3A—C2A—H2A	124.6	C7B—C6B—H6B1	108.4
C4A—C3A—C2A	114.8 (8)	N1B—C6B—H6B2	108.4
C4A—C3A—H3A	122.6	C7B—C6B—H6B2	108.4
C2A—C3A—H3A	122.6	H6B1—C6B—H6B2	107.5
C3A—C4A—C5A	132.7 (7)	O2B—C7B—C6B	111.0 (6)
C3A—C4A—S1A	111.3 (7)	O2B—C7B—C8B	102.7 (5)
C5A—C4A—S1A	116.0 (6)	C6B—C7B—C8B	111.7 (6)
O1A—C5A—N1A	120.8 (8)	O2B—C7B—H7B	110.4
O1A—C5A—C4A	120.5 (8)	C6B—C7B—H7B	110.4
N1A—C5A—C4A	118.7 (7)	C8B—C7B—H7B	110.4
N1A—C6A—C7A	110.4 (6)	N2B—C8B—C7B	101.2 (5)
N1A—C6A—H6A1	109.6	N2B—C8B—H8B1	111.5
C7A—C6A—H6A1	109.6	C7B—C8B—H8B1	111.5
N1A—C6A—H6A2	109.6	N2B—C8B—H8B2	111.5
C7A—C6A—H6A2	109.6	C7B—C8B—H8B2	111.5
H6A1—C6A—H6A2	108.1	H8B1—C8B—H8B2	109.3
O2A—C7A—C6A	109.4 (5)	O3B—C9B—N2B	128.6 (8)
O2A—C7A—C8A	104.3 (5)	O3B—C9B—O2B	121.8 (7)
C6A—C7A—C8A	113.4 (6)	N2B—C9B—O2B	109.6 (7)
O2A—C7A—H7A	109.9	C11B—C10B—C15B	117.5 (7)
C6A—C7A—H7A	109.9	C11B—C10B—N2B	121.1 (7)
C8A—C7A—H7A	109.9	C15B—C10B—N2B	121.1 (7)
N2A—C8A—C7A	103.1 (6)	C12B—C11B—C10B	122.0 (7)
N2A—C8A—H8A1	111.1	C12B—C11B—H11B	119.0
C7A—C8A—H8A1	111.1	C10B—C11B—H11B	119.0
N2A—C8A—H8A2	111.1	C11B—C12B—C13B	120.2 (7)
C7A—C8A—H8A2	111.1	C11B—C12B—H12B	119.9
H8A1—C8A—H8A2	109.1	C13B—C12B—H12B	119.9
O3A—C9A—O2A	120.6 (8)	C12B—C13B—C14B	120.2 (7)
O3A—C9A—N2A	128.2 (8)	C12B—C13B—N3B	120.7 (7)
O2A—C9A—N2A	111.1 (7)	C14B—C13B—N3B	119.1 (7)
C15A—C10A—C11A	120.9 (8)	C13B—C14B—C15B	120.2 (7)
C15A—C10A—N2A	121.0 (7)	C13B—C14B—H14B	119.9
C11A—C10A—N2A	118.1 (7)	C15B—C14B—H14B	119.9
C10A—C11A—C12A	119.6 (8)	C10B—C15B—C14B	119.9 (7)
C10A—C11A—H11A	120.2	C10B—C15B—H15B	120.0
C12A—C11A—H11A	120.2	C14B—C15B—H15B	120.0
C13A—C12A—C11A	120.2 (8)	N3B—C16B—C17B	111.0 (8)
C13A—C12A—H12A	119.9	N3B—C16B—H16C	109.4
C11A—C12A—H12A	119.9	C17B—C16B—H16C	109.4
C12A—C13A—C14A	118.6 (8)	N3B—C16B—H16D	109.4
C12A—C13A—N3A	120.5 (8)	C17B—C16B—H16D	109.4
C14A—C13A—N3A	120.8 (7)	H16C—C16B—H16D	108.0
C15A—C14A—C13A	121.4 (8)	O4B—C17B—C16B	108.2 (7)
C15A—C14A—H14A	119.3	O4B—C17B—H17C	110.1
C13A—C14A—H14A	119.3	C16B—C17B—H17C	110.1
C10A—C15A—C14A	119.3 (8)	O4B—C17B—H17D	110.1
C10A—C15A—H15A	120.3	C16B—C17B—H17D	110.1
C14A—C15A—H15A	120.3	H17C—C17B—H17D	108.4
N3A—C16A—C17A	110.6 (8)	O4B—C18B—C19B	114.2 (7)
N3A—C16A—H16A	109.5	O4B—C18B—H18C	108.7
C17A—C16A—H16A	109.5	C19B—C18B—H18C	108.7
N3A—C16A—H16B	109.5	O4B—C18B—H18D	108.7
C17A—C16A—H16B	109.5	C19B—C18B—H18D	108.7
H16A—C16A—H16B	108.1	H18C—C18B—H18D	107.6
O4A—C17A—C16A	109.3 (7)	O5B—C19B—N3B	123.4 (8)
O4A—C17A—H17A	109.8	O5B—C19B—C18B	118.8 (8)
C16A—C17A—H17A	109.8	N3B—C19B—C18B	117.7 (8)
O4A—C17A—H17B	109.8	C5A—N1A—C6A	118.4 (7)
C16A—C17A—H17B	109.8	C5A—N1A—H1A	120.8
H17A—C17A—H17B	108.3	C6A—N1A—H1A	120.8
O4A—C18A—C19A	116.1 (8)	C9A—N2A—C10A	126.5 (7)
O4A—C18A—H18A	108.3	C9A—N2A—C8A	111.0 (6)
C19A—C18A—H18A	108.3	C10A—N2A—C8A	122.5 (6)
O4A—C18A—H18B	108.3	C19A—N3A—C13A	120.9 (8)
C19A—C18A—H18B	108.3	C19A—N3A—C16A	121.8 (7)
H18A—C18A—H18B	107.4	C13A—N3A—C16A	117.3 (7)
O5A—C19A—N3A	121.9 (8)	C5B—N1B—C6B	122.7 (8)
O5A—C19A—C18A	120.5 (8)	C5B—N1B—H1B	118.7
N3A—C19A—C18A	117.6 (8)	C6B—N1B—H1B	118.7
C2B—C1B—S1B	113.9 (6)	C9B—N2B—C10B	126.6 (7)
C2B—C1B—Cl1B	125.4 (7)	C9B—N2B—C8B	111.3 (6)
S1B—C1B—Cl1B	120.6 (5)	C10B—N2B—C8B	121.9 (6)
C1B—C2B—C3B	111.7 (8)	C19B—N3B—C13B	121.8 (7)
C1B—C2B—H2B	124.1	C19B—N3B—C16B	123.4 (7)
C3B—C2B—H2B	124.1	C13B—N3B—C16B	114.7 (7)
C4B—C3B—C2B	113.3 (8)	C9A—O2A—C7A	109.8 (6)
C4B—C3B—H3B	123.3	C17A—O4A—C18A	110.9 (8)
C2B—C3B—H3B	123.3	C9B—O2B—C7B	109.4 (5)
C3B—C4B—C5B	132.1 (8)	C17B—O4B—C18B	110.3 (7)
C3B—C4B—S1B	110.3 (6)	C1A—S1A—C4A	91.2 (4)
C5B—C4B—S1B	117.5 (6)	C1B—S1B—C4B	90.7 (4)
			
Cl1A—C1A—C2A—C3A	−179.5 (6)	C15A—C10A—N2A—C9A	−18.4 (11)
S1A—C1A—C2A—C3A	−0.4 (9)	C11A—C10A—N2A—C9A	161.4 (8)
C1A—C2A—C3A—C4A	0.2 (11)	C15A—C10A—N2A—C8A	160.6 (7)
C2A—C3A—C4A—C5A	180.0 (9)	C11A—C10A—N2A—C8A	−19.5 (10)
C2A—C3A—C4A—S1A	0.1 (10)	C7A—C8A—N2A—C9A	−1.0 (8)
C3A—C4A—C5A—O1A	172.0 (10)	C7A—C8A—N2A—C10A	179.8 (6)
S1A—C4A—C5A—O1A	−8.1 (12)	O5A—C19A—N3A—C13A	−4.1 (12)
C3A—C4A—C5A—N1A	−8.0 (15)	C18A—C19A—N3A—C13A	177.6 (8)
S1A—C4A—C5A—N1A	171.9 (6)	O5A—C19A—N3A—C16A	175.3 (9)
N1A—C6A—C7A—O2A	−67.0 (8)	C18A—C19A—N3A—C16A	−2.9 (12)
N1A—C6A—C7A—C8A	177.1 (6)	C12A—C13A—N3A—C19A	−117.5 (9)
O2A—C7A—C8A—N2A	5.4 (7)	C14A—C13A—N3A—C19A	67.9 (10)
C6A—C7A—C8A—N2A	124.3 (6)	C12A—C13A—N3A—C16A	63.0 (11)
C15A—C10A—C11A—C12A	−2.2 (13)	C14A—C13A—N3A—C16A	−111.6 (10)
N2A—C10A—C11A—C12A	178.0 (8)	C17A—C16A—N3A—C19A	−19.1 (12)
C10A—C11A—C12A—C13A	1.1 (13)	C17A—C16A—N3A—C13A	160.4 (7)
C11A—C12A—C13A—C14A	1.0 (12)	O1B—C5B—N1B—C6B	10.8 (13)
C11A—C12A—C13A—N3A	−173.7 (8)	C4B—C5B—N1B—C6B	−166.0 (7)
C12A—C13A—C14A—C15A	−2.0 (12)	C7B—C6B—N1B—C5B	−106.8 (9)
N3A—C13A—C14A—C15A	172.7 (8)	O3B—C9B—N2B—C10B	7.6 (12)
C11A—C10A—C15A—C14A	1.2 (12)	O2B—C9B—N2B—C10B	−176.3 (6)
N2A—C10A—C15A—C14A	−179.0 (7)	O3B—C9B—N2B—C8B	−168.0 (8)
C13A—C14A—C15A—C10A	1.0 (12)	O2B—C9B—N2B—C8B	8.1 (8)
N3A—C16A—C17A—O4A	54.0 (11)	C11B—C10B—N2B—C9B	−157.4 (7)
O4A—C18A—C19A—O5A	173.5 (8)	C15B—C10B—N2B—C9B	29.2 (11)
O4A—C18A—C19A—N3A	−8.2 (11)	C11B—C10B—N2B—C8B	17.8 (11)
S1B—C1B—C2B—C3B	−1.8 (10)	C15B—C10B—N2B—C8B	−155.6 (7)
Cl1B—C1B—C2B—C3B	−179.5 (6)	C7B—C8B—N2B—C9B	−20.0 (8)
C1B—C2B—C3B—C4B	1.1 (11)	C7B—C8B—N2B—C10B	164.2 (6)
C2B—C3B—C4B—C5B	176.6 (8)	O5B—C19B—N3B—C13B	0.8 (12)
C2B—C3B—C4B—S1B	0.1 (10)	C18B—C19B—N3B—C13B	−177.4 (7)
C3B—C4B—C5B—O1B	−169.2 (9)	O5B—C19B—N3B—C16B	−179.3 (9)
S1B—C4B—C5B—O1B	7.1 (11)	C18B—C19B—N3B—C16B	2.5 (12)
C3B—C4B—C5B—N1B	7.7 (13)	C12B—C13B—N3B—C19B	114.8 (9)
S1B—C4B—C5B—N1B	−176.0 (6)	C14B—C13B—N3B—C19B	−67.9 (10)
N1B—C6B—C7B—O2B	−59.1 (9)	C12B—C13B—N3B—C16B	−65.2 (10)
N1B—C6B—C7B—C8B	−173.0 (6)	C14B—C13B—N3B—C16B	112.2 (9)
O2B—C7B—C8B—N2B	23.1 (7)	C17B—C16B—N3B—C19B	18.5 (13)
C6B—C7B—C8B—N2B	142.1 (6)	C17B—C16B—N3B—C13B	−161.5 (8)
C15B—C10B—C11B—C12B	1.5 (11)	O3A—C9A—O2A—C7A	−172.5 (7)
N2B—C10B—C11B—C12B	−172.1 (7)	N2A—C9A—O2A—C7A	8.0 (8)
C10B—C11B—C12B—C13B	−0.8 (13)	C6A—C7A—O2A—C9A	−129.8 (6)
C11B—C12B—C13B—C14B	−0.7 (12)	C8A—C7A—O2A—C9A	−8.2 (8)
C11B—C12B—C13B—N3B	176.6 (7)	C16A—C17A—O4A—C18A	−67.0 (11)
C12B—C13B—C14B—C15B	1.4 (11)	C19A—C18A—O4A—C17A	44.0 (11)
N3B—C13B—C14B—C15B	−175.9 (7)	O3B—C9B—O2B—C7B	−175.1 (6)
C11B—C10B—C15B—C14B	−0.8 (10)	N2B—C9B—O2B—C7B	8.5 (7)
N2B—C10B—C15B—C14B	172.8 (7)	C6B—C7B—O2B—C9B	−139.8 (6)
C13B—C14B—C15B—C10B	−0.6 (11)	C8B—C7B—O2B—C9B	−20.3 (7)
N3B—C16B—C17B—O4B	−53.3 (11)	C16B—C17B—O4B—C18B	68.4 (10)
O4B—C18B—C19B—O5B	−167.4 (7)	C19B—C18B—O4B—C17B	−46.9 (9)
O4B—C18B—C19B—N3B	10.9 (10)	C2A—C1A—S1A—C4A	0.4 (7)
O1A—C5A—N1A—C6A	−4.0 (13)	Cl1A—C1A—S1A—C4A	179.5 (5)
C4A—C5A—N1A—C6A	176.0 (6)	C3A—C4A—S1A—C1A	−0.2 (7)
C7A—C6A—N1A—C5A	−171.1 (7)	C5A—C4A—S1A—C1A	179.8 (6)
O3A—C9A—N2A—C10A	−4.6 (13)	C2B—C1B—S1B—C4B	1.6 (7)
O2A—C9A—N2A—C10A	174.9 (6)	Cl1B—C1B—S1B—C4B	179.4 (5)
O3A—C9A—N2A—C8A	176.3 (8)	C3B—C4B—S1B—C1B	−0.9 (7)
O2A—C9A—N2A—C8A	−4.2 (8)	C5B—C4B—S1B—C1B	−178.0 (6)

### Hydrogen-bond geometry (Å, º)

**Table:**

*D*—H···*A*	*D*—H	H···*A*	*D*···*A*	*D*—H···*A*
N1*A*—H1*A*···O3*B*	0.86	2.16	3.008 (11)	169
N1*B*—H1*B*···O3*A*	0.86	2.22	3.016 (11)	153
C3*A*—H3*A*···O3*B*	0.93	2.48	3.357 (11)	157
C6*B*—H6*B*1···O5*A*^i^	0.97	2.41	3.227 (10)	141
C7*A*—H7*A*···O5*B*^ii^	0.98	2.41	3.288 (8)	149
C8*A*—H8*A*2···O1*B*^iii^	0.97	2.52	3.459 (11)	163
C8*B*—H8*B*1···O1*A*^iv^	0.97	2.15	2.961 (12)	140

Symmetry codes: (i) *x*−1, *y*, *z*; (ii) *x*+1, *y*, *z*; (iii) *x*, *y*+1, *z*; (iv) *x*, *y*−1, *z*.
